# Corticomuscular Coherence for Upper Arm Flexor and Extensor Muscles During Isometric Exercise and Cyclically Isokinetic Movement

**DOI:** 10.3389/fnins.2019.00522

**Published:** 2019-05-22

**Authors:** Jinbiao Liu, Yixuan Sheng, Jia Zeng, Honghai Liu

**Affiliations:** State Key Laboratory of Mechanical System and Vibration, School of Mechanical Engineering, Shanghai Jiao Tong University, Shanghai, China

**Keywords:** isokinetic, isometric, electroencephalography, electromyography, corticomuscular coherence, flexors, extensors

## Abstract

Cortical-muscular functional coupling reflects the interaction between the cerebral cortex and the muscle activities. Corticomuscular coherence (CMC) has been extensively revealed in sustained contractions of various upper- and lower-limb muscles during static and dynamic force outputs. However, it is not well-understood that the CMC modulation mechanisms, i.e., the relation between a cerebral hemisphere and dynamic motor controlling limbs at constant speeds, such as isokinetic movement. In this paper, we explore the CMC between upper arm flexors/extensors movement and motor cortex during isometric exercise and cyclically isokinetic movement. We also provide further insights of frequency-shift and the neural pathway mechanisms in isokinetic movement by evaluating the coherence between motor cortex and agonistic or antagonistic muscles. This study is the first to investigate the relationship between cortical-muscular functional connections in elbow flexion-extension movement with constant speeds. The result shows that gamma-range coherence for isokinetic movement is greatly increased compared with isometric exercise, and significant CMC is observed in the entire flexion-extension stage regardless the nature of muscles contraction, although dominant synchronization of cortical oscillation and muscular activity resonated in sustained contraction stage principally. Besides, the CMC for extensors and flexors are explicitly consistent in contraction stage during cyclically isokinetic elbow movement. It is concluded that cortical-muscular coherence can be dynamically modulated as well as selective by cognitive demands of the body, and the time-varying mechanisms of the synchronous motor oscillation exist in healthy individuals during dynamic movement.

## Introduction

Functional coupling between cortical oscillations and muscle activity contributes to neuronal communication during motor control (Yang et al., 2016a; van Vilet et al., 2018). Corticomuscular coherence between electroencephalography (EEG) recorded from the scalp and surface electromyography (sEMG) recorded on the skin is thought to play an important role for neuronal communication between central and peripheral sensorimotor systems (Van Wijk et al., 2012). When performing simple muscle contractions, the human sensorimotor cortex typically produces oscillations associated with muscle activity (Yang et al., 2016b). This indicates that corticomuscular coherence reflects the communication in between the motor cortex and the motor neuron pool (Gross et al., 2000; Fletcher and Wennekers, 2016; Maezawa, 2016; Larsen et al., 2017).

Corticomuscular (EEG-EMG) coherence has been extensively studied for the steady-state motor output (static force or precision grip) (Omlor et al., 2007). Most studies focus on exploring the neural mechanisms associated with the ability to stabilize muscle output (Pfurtscheller and Neuper, 1992; Halliday et al., 1998; Kilner et al., 1999; Feige et al., 2000; Mima et al., 2000). There is still lack of information on the modulation of CMC combined with dynamic force, especially the upper arm muscles that change over time and during exercise cycles. For both static and dynamic motor outputs, the cortical-muscle function coupling reflects the interaction between the cerebral cortex and the muscle activities (Omlor et al., 2007).

Previous investigations demonstrated the types of motor task affected the most prominent frequency band of cortical muscular coherence (Gwin and Ferris, 2012a). Oscillations in the beta (15–30 Hz) frequency bands were extensively observed in recording EEG from the primary motor cortex during static force output (Marsden et al., 2000). Significant gamma (31–45 Hz) frequency bands coherence between the sensorimotor cortex and contracting muscle had been reported during dynamic force output (Gwin and Ferris, 2012a). Omlor et al. (2007) assessed CMC during constant and periodically modulated dynamic force production in a given force feedback task. In the constant condition, significant coherence existed in the beta-range. In the periodically dynamic condition, the most obvious coherence occurred in the gamma-range, and significant coherence shifted from beta band to gamma band. Similarly, Gwin and Ferris (2012a) observed distinct CMC between contralateral motor cortex signals and lower limb EMG signals when the subjects performed isometric muscle activations and isotonic exercise (concentric followed by eccentric) of the right ankle and right knee joints separately. Significant coherence was revealed in the beta- and gamma-range for all task types and gamma-range coherence of isotonic exercise was obviously greater than isometric exercise.

Cortical networks may generate resonation at specific frequencies, and the reflections in both resonant frequencies and cortical networks are dynamic and vary with tasks (Marsden et al., 2000). The possibility of this change likely explains the beta-to-gamma shift of corticomuscular coherence for static vs. dynamic tasks. Gwin and Ferris (2012a) suggested that relative changes in proprioception and muscle dynamics may play a role in the shift of significant coherence toward higher frequencies for the dynamic force task compared with the constant force task. Omlor et al. (2007) supported that the higher gamma frequency coherence during dynamic force output might reflect the complex integration of visual and somatosensory information into motion planning. However, Omlor et al. (2007) only performed the lower level of force (4% maximum voluntary contraction) without considering joints rotation and high-level force. The amplitude of CMC tended to increase with the force increasing in static task and dynamic finger moving task (Fu et al., 2014).

Most studies of upper arm focused on fingers, wrists, and elbows in isometric flexion or extension exercise. Lou et al. (2013) observed significant corticomuscular coherence between the left motor cortical area of the EEG channels and extensor digitorum muscle during finger extension exercise, Divekar and John (2013) had established that wrist flexors revealed significantly lower peak beta-CMC levels compared with wrist extensors during high precision isometric wrist flexion and extension tasks. Bayram et al. (2015) observed significant CMC for agonist and antagonist during sustained isometric elbow flexion, and agonistic muscles presented higher magnitude of CMC compared with antagonist muscles. Cremoux et al. (2017) suggested that spinal cord injury (SCI) had an increased muscle co-activation associated with a decreased magnitude of the CMC at lower frequency band with antagonist muscles. Dynamic movement tasks can be performed in different settings, and control changes can be achieved by moving limbs at constant speed in an isokinetic machine (Kallio et al., 2013). Few documents recorded muscle activities during isokinetic exercise which the angular velocity of the muscle contraction keep constant when joint rotated and did not change with increasing exerted force. Quinzi et al. (2018) believed that isokinetic exercise standardized the range of motion and execution speed of different participants, which was an effective solution to evaluate neuromuscular control. Bravo-Esteban et al. (2017) observed the spasticity syndrome was associated with higher 10–60 Hz intramuscular tibialis anterior coherence during fast isokinetic movement. Flexion and extension movement could be regarded as two consecutive stages of the joint for an isokinetic exercise cycle. In our knowledge, there is no study describing direct or indirect information regarding motor control mechanisms of the dynamic changes in corticomuscular coherence of flexors and extensors with the cerebral cortex during elbow continuous cyclically isokinetic movement which means flexors and extensors complete the whole flexion and extension stages, while previous studies have overwhelmingly focused on sustained contractions of various upper- and lower-limb muscles (Yoshida et al., 2017).

The aim of the presented study is to investigate the changes of corticomuscular coherence between motor cortex and upper arm flexor or extensor muscles during isometric exercise and cyclically isokinetic movement, revealing neural pathway mechanisms of elbow flexion-extension stages. It is hypothesized that the significant coherence area of cyclically isokinetic movement in gamma-range would be higher than that of isometric exercise, inspired by Omlor et al. (2007) that dynamic contractions shifted CMC to gamma-range. Furthermore, we followed effect of continuous rotation of joints on muscle with interest, considering the high-level force output at periodic modulation angle. We further hypothesized that corticomuscluar coherence would be greater for flexor muscles than extensor muscles in contraction stage during the isokinetic cycle in that flexors have a stronger corticospinal connection, since flexors has a stronger corticospinal connection than extensors (Bayram et al., 2015). One of the contributions of the proposed research is taking advantaging of the significant coherent area in time-frequency domain rather than frequency-domain for CMC calculation, the area proportion of significant coherence was utilized to improve the quantification of the corticomuscular coherence.

## Materials and Methods

### Subjects

Ten male subjects (aged 21–27) were invited to participate to the experiments. All recruited subjects were self-reported as being right-handed with no history of major limb injury, and none of them had symptoms or signs of neuromuscular diseases. All subjects gave signed informed consent according to the Helsinki Declaration, and all measurements were approved by the local ethics committee.

### Experiment Procedures

**Figure 1:**
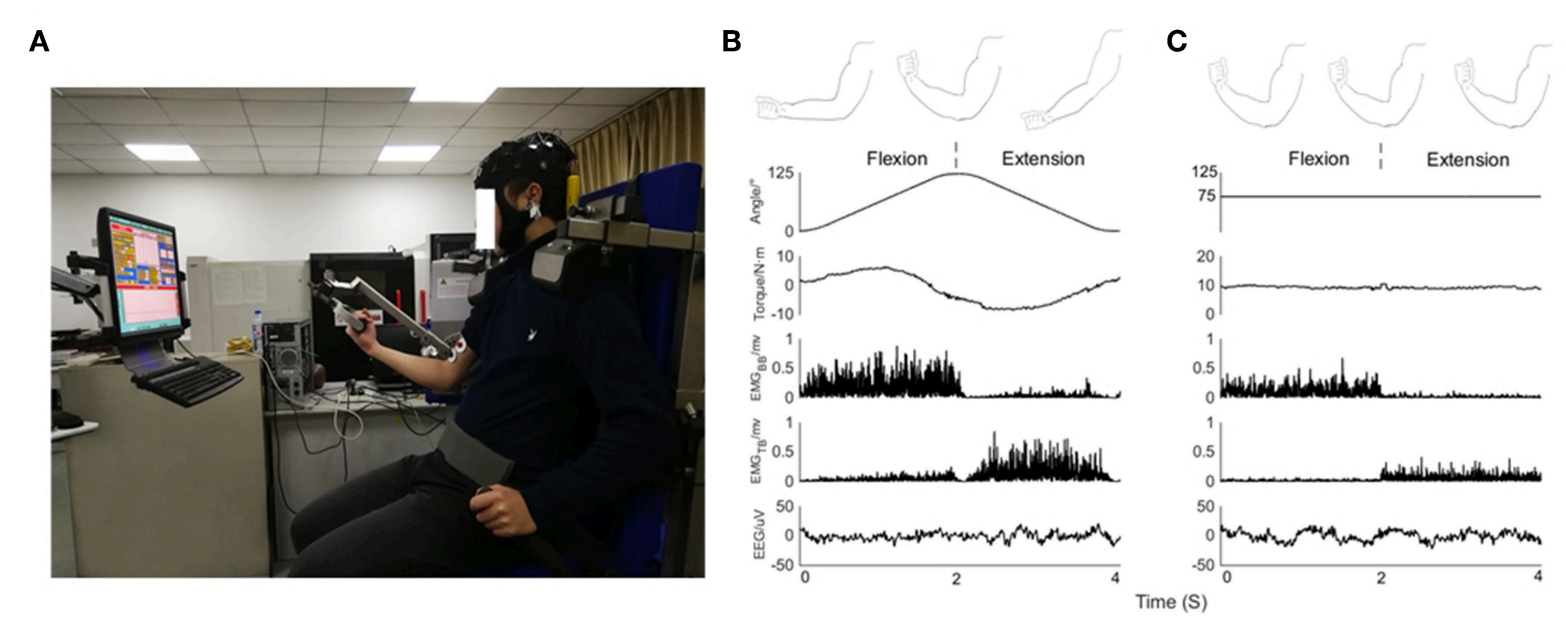
**(A)** Experimental posture of subject on device, EEG signals from 32 scalp positions recorded together with EMG signals. Feedback about the output torque via blue curve and numbers displayed on a monitor in front of the subject. **(B)** From top to bottom, kinematic sketch of upper limb during an elbow joint flexion and extension cycle, rotation angle of elbow joint, output torque, rectified EMG signals of BB muscle and TB muscle and EEG signals recorded in a subject during isokinetic movement condition performed at 60°/s. **(C)** From top to bottom, kinematic sketch of upper limb during flexion and extension of fixed elbow angle, rotation angle of elbow joint, output torque, rectified EMG signals of BB muscle and TB muscle and EEG signals recorded in a subject during isometric exercise condition performed at 75 degrees.

The subjects sat comfortably in the adjustable chair of the Isomed2000 device (D&R Ferstl GmbH, Hemau, Germany) as shown in Figure 1A. The trunk of the subject was fixed with a belt, and two shoulders were locked with cushions to stabilize the posture during the experiments. Meanwhile, the right elbow was supported by a fulcrum and adjusted to the most comfortable position to facilitate flexion and extension. The subject's upper arm and adapter (mechanical arm) remained in a relatively horizontal orientation, and subject held the handle with right hand. The force exerted by the subject could be recorded and transmitted to the computer monitor in front of him through a dynamometer. Subject received feedback reminders regarding the output torque via blue curves and numbers on the screen. Dynamometer could apply two training modes, isokinetic movement and isometric exercise.

The subjects performed experimental tasks under two different experimental scenarios:

1. Isokinetic movement condition: the elbow moved periodically in the range of 0 degrees to 125 degrees, dynamometer performed at constant 60°/s velocity. Subject completed flexion and extension exercises with 30% cyclic mean voluntary torque (CMVT) determined prior to the experiment (Figure 1B).
2. Isometric exercise condition: the elbow was fixed at a relative angle of 75 degrees. Subject exerted 30% maximum voluntary torque (MVT) determined prior to the experiment (Figure 1C).

The relative angle refers to the condition when a subject is sitting in a normal posture where 0 degrees represents the lowest position in the natural drop of the arm and the angle increases in a clockwise direction. Isokinetic movement is a periodic uniform motion without constant torque output during the joint rotation. CMVT means the average torque value at each angle in the isokinetic cycle, which can be observed in real time through the computer screen. For isometric exercise task, subjects maintained 30% MVT for 4s as a trial, performing 2s of flexion followed by 2s extension during the 4s isometric trials, then rest interval of 5s between the trials, they completed a series of 40 trials. For isokinetic movement task, subjects performed both elbow flexion and extension as a trial that was a continuous periodic movement without time interval, the output torque was 30% CMVT determined prior to the experiment, single trial duration was set to 4s (2s flexion and 2s extension), they completed a series of 40 trials and rested for 5s in each trial. During the experiment, subjects were asked to avoid any other actions and to focus on torque feedback. Besides, the experimental paradigm design also reduced the possibility of fatigue. In order to evaluate muscle fatigue, referring to the previous studies (Yoshitake et al., 2001; Felici et al., 2009; Guo et al., 2017), we extracted the characteristics of time (mean square root, RMS) and frequency (median frequency, MDF) information of EMG as fatigue parameters in the preliminary experiment. The performance of muscle fatigue on EMG is that the power spectrum shifts left, the median frequency decreases, and the RMS of signal energy increases. We use these characteristics to determine whether muscles are fatigued or not. The variations of fatigue parameters (RMS, MDF) are expressed as % of the baseline value (mean value of the first trial). The values are group averaged across all subjects.

### Data Recording

The Enobio 32-channel system (Neuroelectrics, Barcelona, Spain) is used to acquire EEG according to the international 10/10 system at a sampling rate of 500 Hz with band pass filter 0.540–40 Hz (Angulo-Sherman et al., 2017). We used the user-defined mode of the Enobio device to assign electrodes position in the head cap for each electrode. Some default electrodes were replaced by more suitable ones. The reference electrode was placed at right earlobe. Impedance of the EEG electrodes was kept below 5 Kohm during the recording. We applied a high-pass at 0.5 Hz (zero-lag, 4th order Butterworth filter) to the EEG signals to remove drift, and eliminated EEG signals exhibiting substantial noise during acquisition process (Gwin and Ferris, 2012b). EEGLAB (an open source Matlab toolbox for processing electrophysiological data) based scripts in Matlab (The Mathworks, Natick, MA) was used for all processing and analysis.

The wireless multi-channel sEMG acquisition device (Jiaopu Tech Ltd, China) was applied to capture the sEMG signals. The EMG signals were recorded from biceps brachia (BB) muscle and triceps brachii (TB) muscle. The electrodes used electrodes with a diameter of 10 mm in bipolar configuration and inter-electrode spacing of 10 mm. The signals were processed with high-pass filtered at 3 Hz (zero-lag, 4th order Butterworth filter), sampled at 1,000 Hz, and A/D converted with 12-bit resolution.

Torque and angle data were simultaneously recorded through Isomed2000 device with 500 Hz sampling rate. EEG, EMG, and mechanical signals were collected synchronously by trigger and all data analysis were performed offline using MATLAB R2014b with the sampling frequency of 500 Hz.

### Data Analysis

#### Data Preprocessing

EMG signals were rectified, as full wave rectification was known to provide the temporal pattern of grouped firing motor units (Boonstra, 2010; Halliday and Farmer, 2010; Ward et al., 2013; Dakin et al., 2014; Farmer and Halliday, 2014). Independent component analysis (ICA) algorithm based on EEGLab software was used to remove artifacts in EEG signals caused by blinking or eye movement. ADJUST plug-in in EEGLAB removes the noise components of EEG signals after ICA. Based on experience and plug-in identification, the noise related components are removed. ICA is run several times and the most representative components are taken as the final result to ensure the stability of ICA decomposition. There was no significant difference in the number of rejected IC across conditions [*F*_(1,18)_ = 0.228, *p* = 0.639]. For both tasks, simultaneously recorded EEG and EMG signals of all experimental trials of each subject. Every segment corresponded to the whole flexion and extension stages (Figures 1B,C showed: 0–2 s corresponded to flexion stage, 2–4 s corresponded to extension stage). After the preprocessing stage, for isometric condition, the number of trials remaining is 38.2 ± 0.74; for isokinetic condition, the number of trials remaining is 37.6 ± 0.92. Whatever the task condition, the number of trials used for further analysis was no significant difference across conditions [*F*_(1,18)_ = 2.314, *p* = 0.146].

#### Continuous Wavelets Transform Based Coherence Estimate

Wavelet analysis has been devised to analyze signals with rapidly changing spectra. Continuous wavelets transform (CWT) uses time-scale function to analyze signals and constructs wavelet basis by translation and scaling, since wavelet have both time shifting and multi-scale resolution, time-frequency domain analysis can be performed simultaneously. Let $X={\left[x\left(t_{r}\right)\right]}_{r=1}^{T}$ and $Y={\left[y\left(t_{r}\right)\right]}_{r=1}^{T}$ denote two random time series of length *T* observed at regular time points *t*_*r*_. The CWT of signal *X* (or *Y*) at scale *c* > 0 and time *s* is defined as

$$CWT_{X}\left(c,s\right)=\sum_{r=1}^{T}x(t_{r})\cdot \psi_{c,s}^{*}(t_{r})$$

Where ^*^ denotes complex conjugate,

$$\begin{array}{l} \psi_{c,s}\left(t_{r}\right)=\frac{1}{\sqrt{c}}\psi \left(\frac{t_{r}-s}{c}\right) \end{array}$$

and ψ(*t*_*r*_) is called the mother wavelet which should satisfy a number of selection criteria and admissibility condition (Madhavan, 2003; Zhan et al., 2006). CWT is usually seen as a time–frequency representation by converting the scale parameter *c* to frequency *w*. Usually defined a specific frequency *w*_0_ to represent the central frequency location of the energy ψ in Fourier domain, and the relationship between the frequency and scale is achieved by *w* = *w*_0_/*c*. The CWT at time *s* and frequency *w*_0_ can be expressed as Madhavan (2003)

$$\begin{array}{l} CWT_{X}\left(w,s\right)=\overset{T}{\underset{r=1}{\sum }}x\left(t_{r}\right)\cdot \sqrt{\frac{w}{w_{0}}}\cdot \psi \left(\frac{w}{w_{0}}\left(t_{r}-s\right)\right) \end{array}$$

Morlet wavelet is one of the most commonly used wavelets in practice, defined as Kronlandmartinet et al. (1987)

$$\begin{array}{l} \psi \left(s\right)=\frac{1}{\pi^{1/4}}\cdot e^{iw_{0}s}\cdot e^{-s^{2}/2} \end{array}$$

Where *w*_0_ is the central frequency of ψ. Morlet wavelet analysis is a simple and suitable wavelet for spectral estimations, and has an excellent balance between the localization of time and frequency (Lachaux et al., 2002; Grinsted et al., 2004; Ombao and Van Bellegem, 2008; Kha et al., 2018). Besides, morlet wavelets are non-orthogonal and exponential complex wavelet adjusted by Gaussian, which can achieve smooth and continuous wavelet amplitudes. We took *w*_0_ = 6 as it was a good choice in wavelet analysis of neurophysiological signals in this paper.

The CWT based coherence at frequency *w* and time *s* between the time series *X* and *Y* is defined as the following equation (Zhan et al., 2006):

$$\begin{array}{l} R_{XY}^{2}\left(w,s\right)=\frac{{\left|S_{XY}\left(w,s\right)\right|}^{2}}{S_{X}\left(w,s\right)S_{Y}\left(w,s\right)} \end{array}$$

where *S*_*XY*_(*w, s*) is the wavelet cross-spectrum (WCS) between *X* and *Y*, *S*_*X*_(*w, s*) and *S*_*Y*_(*w, s*) are the wavelet auto-spectrum (WAS) of the two signals defined, respectively as Bigot et al. (2011).

$$\begin{array}{l} S_{XY}\left(w,s\right)=\mathbb{P}\left(CWT_{X}\left(w,s\right)\cdot CWT_{Y}^{*}\left(w,s\right)\right)\\ {\;S}_{X}\left(w,s\right)=\mathbb{P}{\left|CWT_{X}\left(w,s\right)\right|}^{2}\\ {\;S}_{Y}\left(w,s\right)=\mathbb{P}{\left|CWT_{Y}\left(w,s\right)\right|}^{2} \end{array}$$

where ℙℤ denotes the expectation of a random variable *Z*.

According to law of large numbers, for one observed time series consisting of *n* repeated segments ${\left(X_{u}\right)}_{u=1,\ldots ,n}={\left({\left[x_{u}(t_{r})\right]}_{r=1}^{T}\right)}_{u=1,\ldots ,n}$ and ${\left(Y_{u}\right)}_{u=1,\ldots ,n}={\left({\left[y_{u}(t_{r})\right]}_{r=1}^{T}\right)}_{u=1,\ldots ,n}$, we considered as *n* independent realizations of the stochastic processes *X* and *Y*, and then we could estimate the WCS and WAS of the two series by empirical WCS defined as Bigot et al. (2011).

$$\begin{array}{l} {Ŝ}_{XY}\left(w,s\right)=\frac{\sum_{u=1}^{n}CWT_{X_{u}}\left(w,s\right)\cdot CWT_{Y_{u}}^{*}\left(w,s\right)}{n}\\ {\;Ŝ}_{X}\left(w,s\right)=\frac{\sum_{u=1}^{n}{{|CWT}_{X_{u}}\left(w,s\right)|}^{2}}{n}\\ {\;Ŝ}_{Y}\left(w,s\right)=\frac{\sum_{u=1}^{n}{{|CWT}_{Y_{u}}\left(w,s\right)|}^{2}}{n} \end{array}$$

and corresponding empirical wavelet coherence is as follows

$$\begin{array}{l} {\hat{R}}_{XY}^{2}\left(w,s\right)=\frac{{\left|\sum_{u=1}^{n}CWT_{X_{u}}\left(w,s\right)\cdot CWT_{Y_{u}}^{*}\left(w,s\right)\right|}^{2}}{\left(\sum_{u=1}^{n}{{|CWT}_{X_{u}}\left(w,s\right)|}^{2}\right)\left(\sum_{u=1}^{n}{{|CWT}_{Y_{u}}\left(w,s\right)|}^{2}\right)} \end{array}$$

The coherence $R_{XY}^{2}\left(w,s\right)$ could be seen as the limit of ${\hat{R}}_{XY}^{2}\left(w,s\right)$ when the number of trials *n* tends to infinity. For frequency *w* and time, the time-frequency based coherence is normalized and satisfied 0 to 1, where 1 indicates an ideal correlation between two signals and 0 indicates a total absence of association.

The method proposed in the literature of obtaining a threshold to detect significant values of coherence is based on the assumption that the two series have independent Gaussian distribution (Gish and Cochran, 1988). Under the null hypothesis *H*_0_, confidence interval is 1−α, the thresholds *r*_α_ is given by

$$\begin{array}{l} r_{\alpha }=1-\alpha^{\frac{1}{n-1}},\;0\ll \alpha \ll 1 \end{array}$$

**Figure 2:**
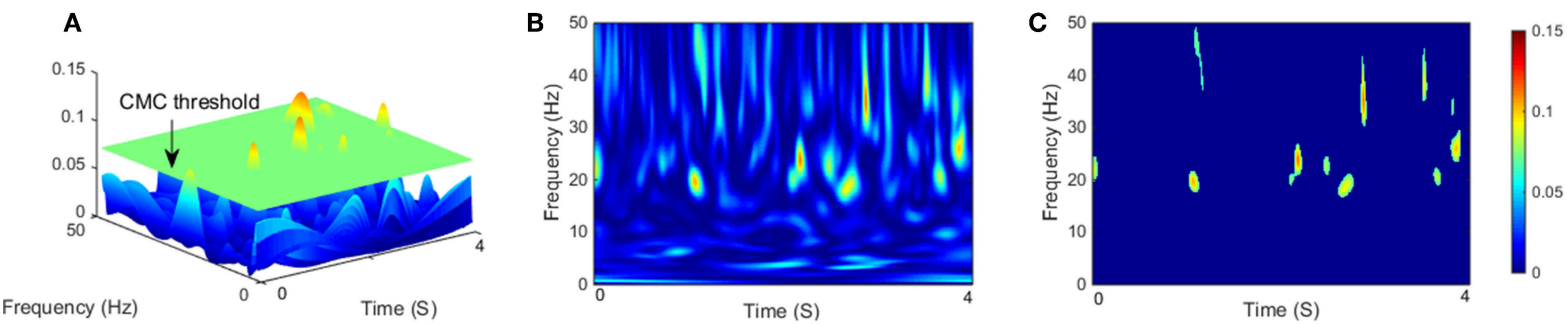
**(A)** Three-dimensional representation of CMC on the time-frequency plane with a segment length of 4 s, the green flat indicated threshold of significant coherence, and the magnitude of CMC was quantified as the area exceeded the threshold level. **(B)** Two-dimensional empirical wavelet coherence ${\hat{R}}_{XY}^{2}\left(w,s\right)$ using CWT on the time–frequency plane. **(C)** Two-dimensional significant coherence ${\hat{R}}_{SCS}^{2}\left(w,s\right)$, represents significant areas of **B**.

In this study, the confidence interval was set to 95%, the level α = 5%, value of ${\hat{R}}_{XY}^{2}\left(w,s\right)$ that above the threshold was considered for significant coherence. Before applying the threshold, the magnitude of wavelet coherence was quantified as the area of significant coherence where exceeded the threshold level (Figure 2A). ${\hat{R}}_{XY}^{2}\left(w,s\right)$ as coherence spectra was binned across frequency (0–50 Hz) and time (4 s single segment). The frequency resolution in this study was given 0.2 Hz (Figure 2B). Previous research has typically quantified coherence without temporal resolution (Yoshida et al., 2017). This method is suitable for quantitative analysis of coherence during continuous static muscle contraction since cortical involvement could be assumed to be relatively stable (Hellwig et al., 2001). However, for dynamic movements such as isokinetic exercise, it is more intuitive to consider the time modulation of coherence in each motion cycle. Similar to (Kilner et al., 2000), we defined any points below or equal the threshold of the binary spectra were set to 0, named significant coherence value (SCV) ${\hat{R}}_{SCV}^{2}\left(w,s\right)$, which mean ${\hat{R}}_{XY}^{2}\left(w,s\right)>r_{\alpha }$ (Figure 2C).

Our study required the estimation of significant coherence between EEG and EMG in different frequency bands. We defined *CMC*_*sig*_ that the area proportion of significant coherence in the corresponding frequency bands as following

$$CMC_{sig}=\frac{\mathbb{N}|{\left({\left[{\hat{R}}_{SCV}^{2}\left(w,s\right)\right]}_{w=w_{1}}^{w_{2}}\right)}_{s=1,\ldots ,s_{k}}|}{\mathbb{N}|{\left({\left[{\hat{R}}_{XY}^{2}\left(w,s\right)\right]}_{w=w_{1}}^{w_{2}}\right)}_{s=1,\ldots ,s_{k}}|}$$

where $\mathbb{N}|{\hat{R}}_{SCV}^{2}{(}^{*})|$ denoted the sum of the non-zero point in the SCS, and $\mathbb{N}|{\hat{R}}_{XY}^{2}(*)|$ was the sum of all point in coherence spectra. The point was viewed as one pixel over the frequency-time plane which only as statistics, without considering specific value, *w*_1_ and *w*_2_ denoted the lower and upper limits of frequency band (for beta band, *w*_1_ = 15 *Hz, w*_2_ = 30 *Hz*, and for gamma band, *w*_1_ = 31 *Hz, w*_2_ = 45 *Hz*), *k* was the time series points length of single segment, and then *s*_*k*_ meant the last point of time series in a segment.

### Statistical Analysis

**Figure 3:**
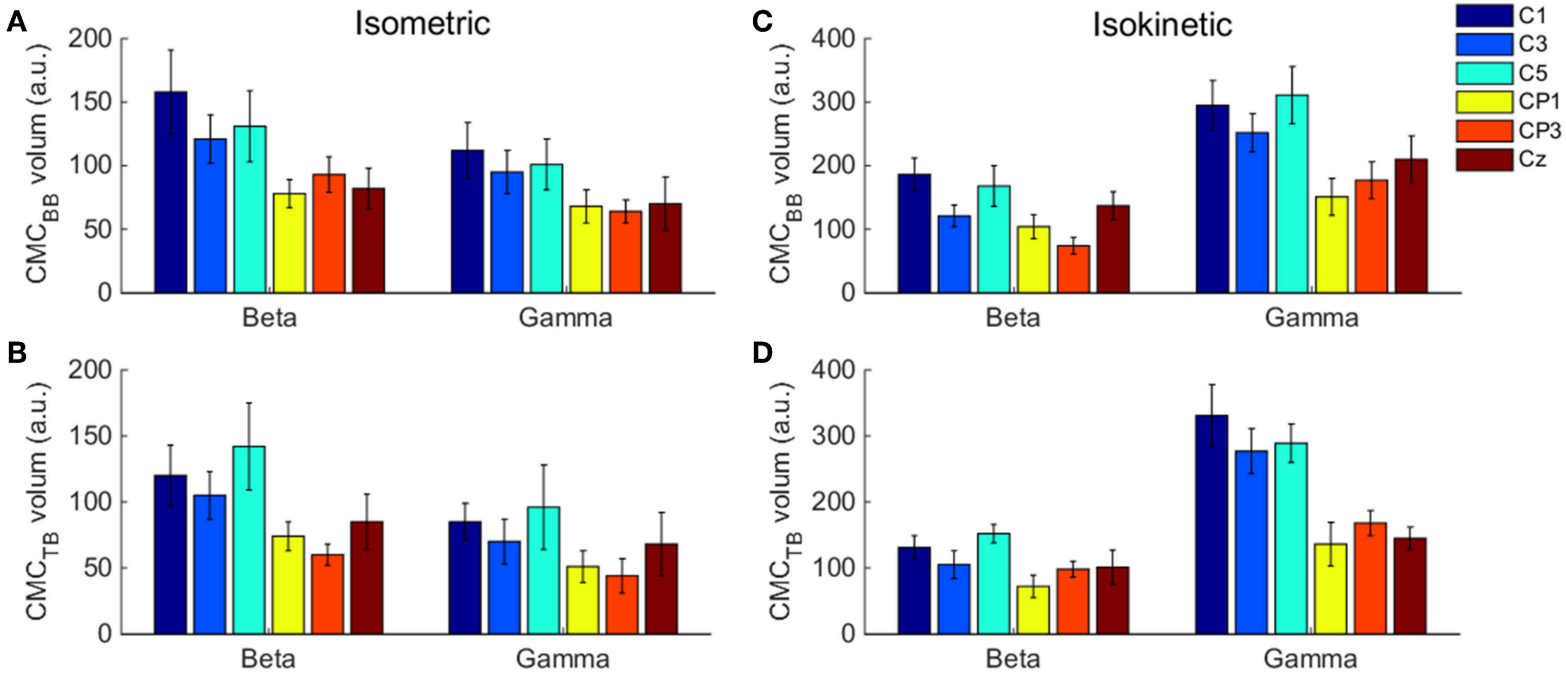
. The volume of significant CMC between each EEG channel and BB and TB muscles in the beta- and gamma-range during isometric exercise **(A,B)** and isokinetic movement **(C,D)**. We denoted the volume of these 6 EEG channels is higher than other channels. Error bars show the standard error of the mean.

To evaluate muscle fatigue in the preliminary experiment, one-way analysis of variance (ANOVA) was performed between the trail after baseline value and the other trials of muscle contraction during isometric and isokinetic tasks, respectively. To estimate any statistical difference on CMC magnitude of subjects between the isometric exercise and cyclically isokinetic movement (flexion-extension stage) condition, CMC magnitude was quantified as volume under the time-frequency plane where CMC was significant (Figure 2A shows the three-dimensional time-frequency plane). This method of magnitude quantification was introduced in previous CMC studies (Cremoux et al., 2017; Maso et al., 2017). The results showed that the volume of significant CMC in C1 and C5 channels was higher than other EEG channels (Figure 3). To compare the volume statistics, we counted the average *CMC*_*sig*_ under each EEG channel. Statistical results indicated that the EEG electrodes showed the significant coherence with rectified EMG only in areas C1, C3, C5, FC1, FC3, FC5, CP1, CP3, CP5, Cz, and the *CMC*_*sig*_ in C1 and C5 were higher than other EEG channels, which were consistent with the result of volume statistics. Accordingly, for each participant and experimental condition, we selected EEG data from the C1 and C5 channel for quantitative analyses, CMC in elbow extensor and flexor muscle groups was obtained by averaging *CMC*_*sig*_ in C1 EEG–BB EMG and C1 EEG–TB EMG and in C5 EEG–BB EMG and C5 EEG–TB EMG, respectively.

We examined how coherence differed in grand average of *CMC*_*sig*_ under C1 and C5 electrodes by performing a three-way ANOVA with the (i) type of task (isometric or isokinetic), (ii) type of frequency bands (beta or gamma), and (iii) muscles (BB or TB) as factors. In order to analysis CMC difference during elbow flexion and extension stages when muscles acted as agonist and antagonist, a one-way ANOVA regarding the *CMC*_*sig*_ value of the flexion and extension stages during isometric exercise and isokinetic movement was calculated. To examine whether there was a significant difference in the coherence between these two muscles and the primary motor cortex during the contraction stage of the isometric exercise and isokinetic movement, we utilized a two-way ANOVA to assess the statistical significance by the grand average of *CMC*_*sig*_ in independent variables frequency (beta or gamma) and muscles (flexors or extensors) at muscles contraction stage of cyclically isokinetic elbow movement and isometric exercise, respectively. The significance level was set at α = 0.05, and Bonferroni correction was used to solve multiple comparison problems. Statistical analysis was performed using SPSS 22.0 (SPSS Inc., Chicago, IL, USA).

## Results

### Pre-Experiment Analysis on Fatigue

Pre-experiment results showed no significant difference in the RMS [*F*_(1,18)_ = 3.297, *p* = 0.086] and MDF [*F*_(1,18)_ = 2.886, *p* = 0.107] between the first and last trail during isometric contraction. For isokinetic task, there was also no significant difference in the RMS [*F*_(1,18)_ = 2.565, *p* = 0.127] and MDF [*F*_(1,18)_ = 1.109, *p* = 0.306]. It was found that the RMS was no significantly increased after all trials, and no significant changes were appeared for MDF, which demonstrated that the muscle was not in fatigue state during the experiment.

### Significant Coherence Analysis of Isometric Exercise and Isokinetic Movement

The volume of significant CMC between each EEG channel and BB and TB muscles in the beta- and gamma-range during isometric exercise and isokinetic movement were shown in Figure 3. In different tasks, muscles and frequency bands, C1 and C5 channels showed higher volume than other channels.

**Figure 4:**
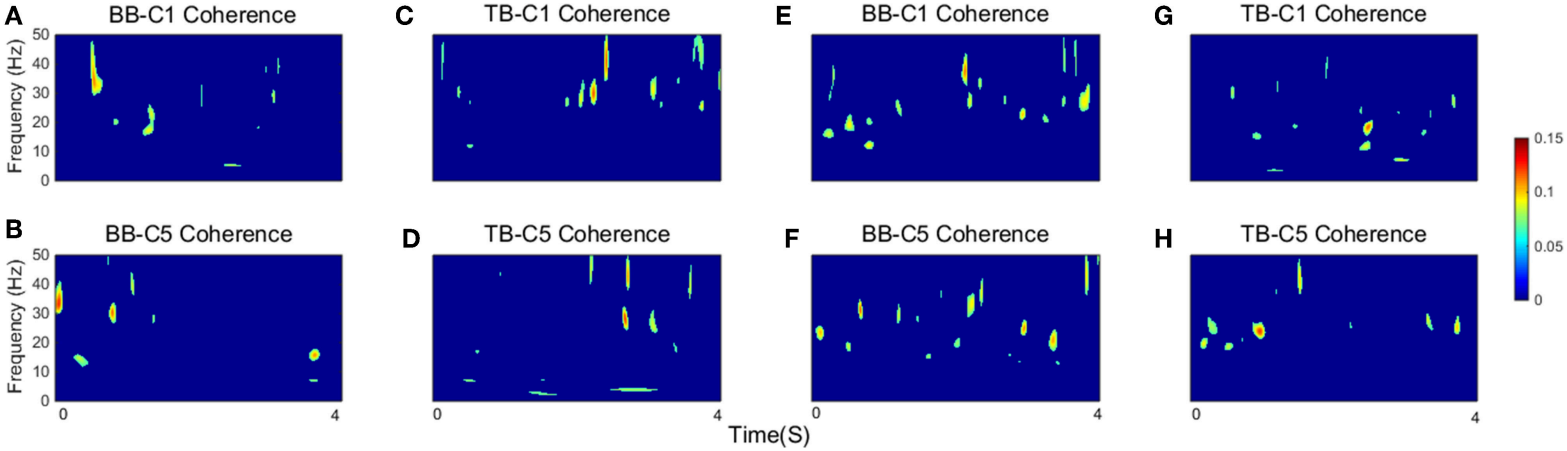
The significant area of coherence between EEG signal from C1 and C5 and EMG signal from the BB and TB muscles of a representative participant. **(A–D)** show SCV for isokinetic movement, **(E–H)** show SCV for isometric exercise, respectively. Confidence level was 95%.

As defined in the previous section, SCV is more intuitive and visualized to consider the time-frequency modulation of coherence. Figure 4 showed significant area of coherence between EEG signal from C1 and C5 and EMG signal from the BB and TB muscles of a representative participant. The corresponding *CMC*_*sig*_ results were listed in Table 1. It showed that the *CMC*_*sig*_ value in gamma-range was higher than in beta-range during isokinetic movement according to BB-C1, BB-C5, TB-C1, and TB-C5 coherence. On the contrary, the *CMC*_*sig*_ value in gamma-range was lower than in beta-range during isometric exercise according to BB-C1, BB-C5, TB-C1, and TB-C5 coherence.

**Table 1:** *CMC*_*sig*_ value of a representative participant according frequency.

**Condition**	**Frequency**	**BB-C1**	**BB-C5**	**TB-C1**	**TB-C5**
Isokinetic	Beta	0.042	0.033	0.046	0.041
	Gamma	0.061	0.050	0.057	0.053
Isometric	Beta	0.073	0.063	0.051	0.066
	Gamma	0.056	0.052	0.035	0.048

**Figure 5:**
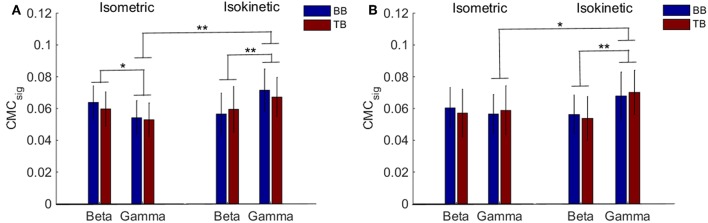
Grand average of the *CMC*_*sig*_ values between EEG signal from C1 and C5 and EMG signal from the BB and TB muscle during isometric exercise and isokinetic movement in the beta- and gamma-range, respectively. **(A,B)** show the *CMC*_*sig*_ values under C1 and C5 electrodes, respectively. We denoted the significance with the star mark. *p < 0.05 and **p < 0.01. Error bars show the standard error of the mean.

Accordingly, we used *CMC*_*sig*_ value to quantitatively analyze coherence and examined how coherence differed in grand average of *CMC*_*sig*_ under C1 and C5 electrodes by performing a three-way ANOVA. In our statistical analysis, we calculated the average values of *CMC*_*sig*_ for two frequency bands (beta and gamma), two upper arm muscles (BB muscle and TB muscle) and two exercise conditions (isometric and isokinetic), respectively. For the *CMC*_*sig*_ values under C1 electrode, as shown in Figure 5A, there were no three-factor significant interaction between frequency bands, muscles and conditions [*F*_(1,72)_ = 0.783, *p* = 0.379] for the average *CMC*_*sig*_value, but a two-factor significant interaction between frequency bands and conditions was revealed [*F*_(1,72)_ = 11.797, *p* = 0.001]. The average value was significantly affected by frequency bands in isometric exercise [*F*_(1,72)_ = 4.137, *p* = 0.046], isokinetic movement [*F*_(1,72)_ = 7.971, *p* = 0.006] and exercise conditions in gamma-range [*F*_(1,72)_ = 15.401, *p* < 0.001]. Neither the muscles and exercise conditions factor [*F*_(1,72)_ = 0.127, *p* = 0.723] nor the frequency bands and muscles factor [*F*_(1,72)_ = 0.697, *p* = 0.152] interacted significantly for the average *CMC*_*sig*_ value. Additionally, for the *CMC*_*sig*_ values under C5 electrode, as shown in Figure 5B, there were also no three-factor significant interaction between frequency bands, muscles and conditions [*F*_(1,72)_ = 0.005, *p* = 0.943] for the average *CMC*_*sig*_value, but a two-factor significant interaction between frequency bands and conditions was revealed [*F*_(1,72)_ = 5.327, *p* = 0.024]. The average value was significantly affected by frequency bands in isokinetic movement [*F*_(1,72)_ = 9.148, *p* = 0.003] and exercise conditions in gamma-range [*F*_(1,72)_ = 6.008, *p* < 0.017]. Neither the muscles and exercise conditions factor [*F*_(1,72)_ = 0.003, *p* = 0.954] nor the frequency bands and muscles factor [*F*_(1,72)_ = 0.609, *p* = 0.438] interacted significantly for the average *CMC*_*sig*_ value. The results indicated that for C1 and C5 electrodes, the coherence of gamma-range in isokinetic movement is significantly higher than isometric exercise, and the coherence of beta-range is significantly lower than gamma-range during the isokinetic movement. The obvious coherence occurred in gamma-range for isokinetic movement, and coherence shifted from beta-range to gamma-range for isometric vs. isokinetic tasks.

### CMC Analysis When Muscles Acted as Agonist and Antagonist

For different tasks, there were potential differences in significant coherence between flexion and extension stages when muscles acted as agonist and antagonist. In order to show this shift in terms of temporal evolution, we investigated the group average time-frequency maps of significant coherence. For the group average, the threshold of significance was calculated using the number of movement cycles completed among all participants during tasks. According to the group average time-frequency maps, we calculated the *CMC*_*sig*_ value by one-way ANOVA. The *CMC*_*sig*_ value of the flexion and extension stages when muscles acted as agonist and antagonist during isometric exercise and isokinetic movement according different frequency could be observed in Table 2. During isokinetic movement, for BB muscle, statistical results show the *CMC*_*sig*_ value of the flexion stage is significantly higher than that of the extension stage in both beta-range (0.048 ± 0.006 vs. 0.045 ± 0.005) and gamma-range (0.055 ± 0.014 vs. 0.049 ± 0.012) according to BB-C1 coherence, whereas for TB muscle, on the contrary, the value of the flexion stage is significantly lower than that of the extension stage in both beta-range (0.038 ± 0.004 vs. 0.040 ± 0.006) and gamma-range (0.043 ± 0.008 vs. 0.050 ± 0.007) according to TB-C1 coherence. The phenomena of C1 and C5 electrodes are basically the same. Significant coherence was increased rapidly at the contraction stage, i.e., during flexion phase of BB muscle and extension phase of TB muscle, but reduced significantly at the relaxation stage, i.e., during extension phase of BB muscle and flexion phase of TB muscle. However, during isometric exercise, there was no significant inclination trend of the *CMC*_*sig*_ value in flexion and extension stages.

**Table 2:** Results of one-way ANOVA on the *CMC*_*sig*_ value of the flexion and extension stages according different conditions and frequency.

**Condition**	**Frequency**	**BB-C1**	**BB-C5**	**TB-C1**	**TB-C5**
Isokinetic	Beta	*F*_(1,238)_ = 16.355, *P* < 0.001	*F*_(1,238)_ = 10.035, *P* = 0.002	*F*_(1,238)_ = 6.249, *P* = 0.013	*F*_(1,238)_ = 5.285, *P* = 0.022
	Gamma	*F*_(1,238)_ = 11.487, *P* = 0.001	*F*_(1,238)_ = 18.496, *P* < 0.001	*F*_(1,238)_ = 21.946, *P* < 0.001	*F*_(1,238)_ = 34.101, *P* < 0.001
Isometric	Gamma	*F*_(1,238)_ = 11.487, *P* = 0.001	*F*_(1,238)_ = 18.496, *P* < 0.001	*F*_(1,238)_ = 21.946, *P* < 0.001	*F*_(1,238)_ = 34.101, *P* < 0.001
	Gamma	*F*_(1,238)_ = 3.032, *P* = 0.083	*F*_(1,238)_ = 0.790, *P* = 0.375	*F*_(1,238)_ = 1.243, *P* = 0.266	*F*_(1,238)_ = 0.209, *P* = 0.648

The results indicated that the coherence of muscle function acting as agonist is significantly higher than that of acting as antagonist during isokinetic movement.

**Figure 6:**
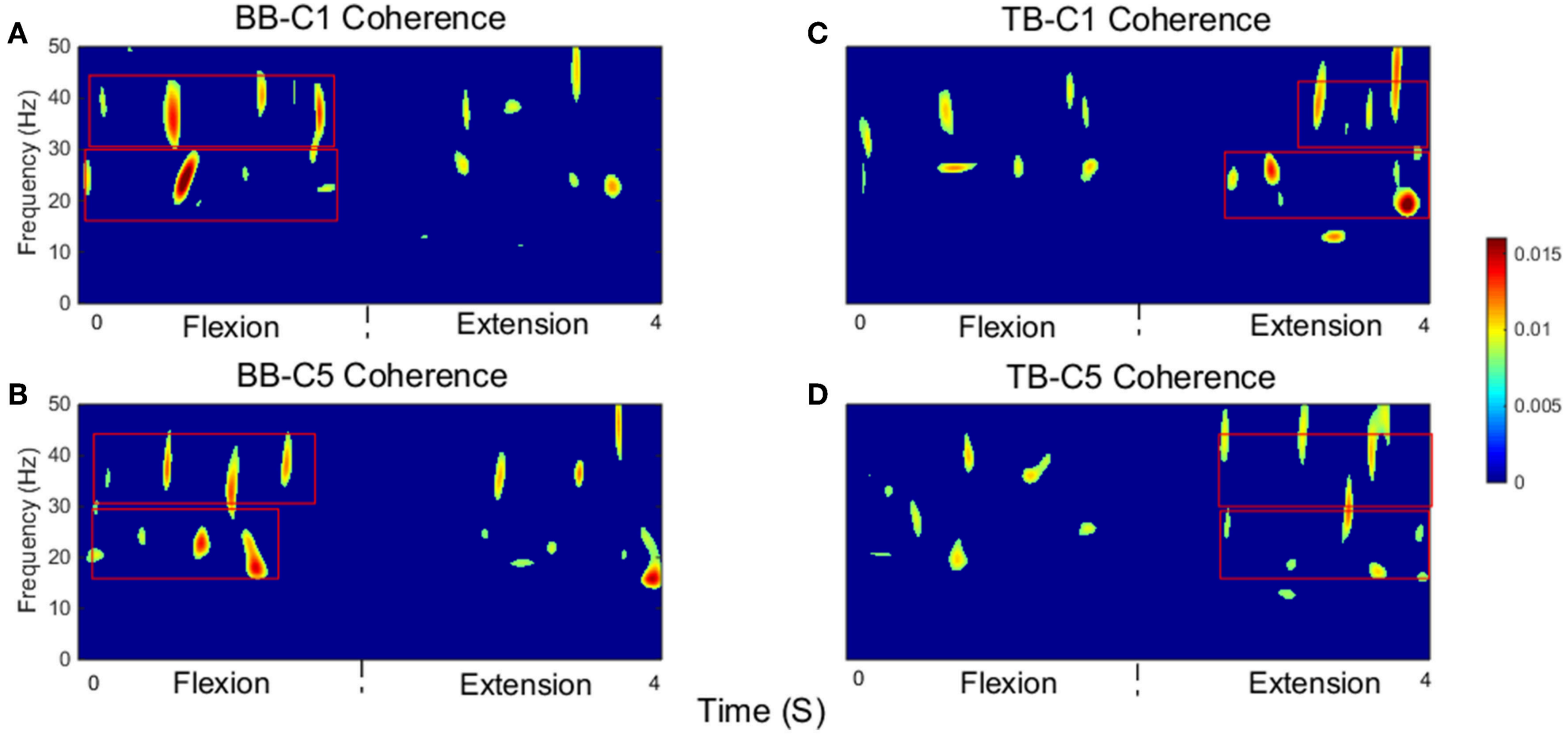
Group average of significant CMC between EEG signal from C1 and C5 and EMG signal from the BB and TB muscles during isokinetic movement. **(A,B)** show SCV for BB-C1 and BB-C5 coherence, **(C,D)** show SCV for TB-C1 and TB-C5 coherence, respectively. Note that red box areas indicate that BB and TB muscles acted as agonist, in beta and gamma bands, respectively. The first 2 s are elbow flexion stage and the last 2 s are elbow extension stage. Confidence level was 95%.

Significant time-frequency maps of isokinetic task were shown in Figure 6, we could clearly see the temporal evolution of coherence shift between BB (Figures 6A,B) and TB (Figures 6C,D) in flexion and extension stages. BB muscles acted as agonist in elbow flexion stage and antagonist in elbow extension stage, while the TB muscle was the opposite. Red box areas in Figure 6 indicated that BB and TB muscles acted as agonist, in beta and gamma bands, respectively. Obviously, the significant coherence existed throughout the flexion-extension stages regardless of muscle function, but when muscles acted as agonist, the significant coherence areas were more widely distributed. The group average time-frequency maps of significant coherence could intuitively illustrate the temporal evolution of coherence in the whole flexion-extension stage.

### Compare the Differences of CMC Between BB and TB Muscles Acting as Agonist

**Figure 7:**
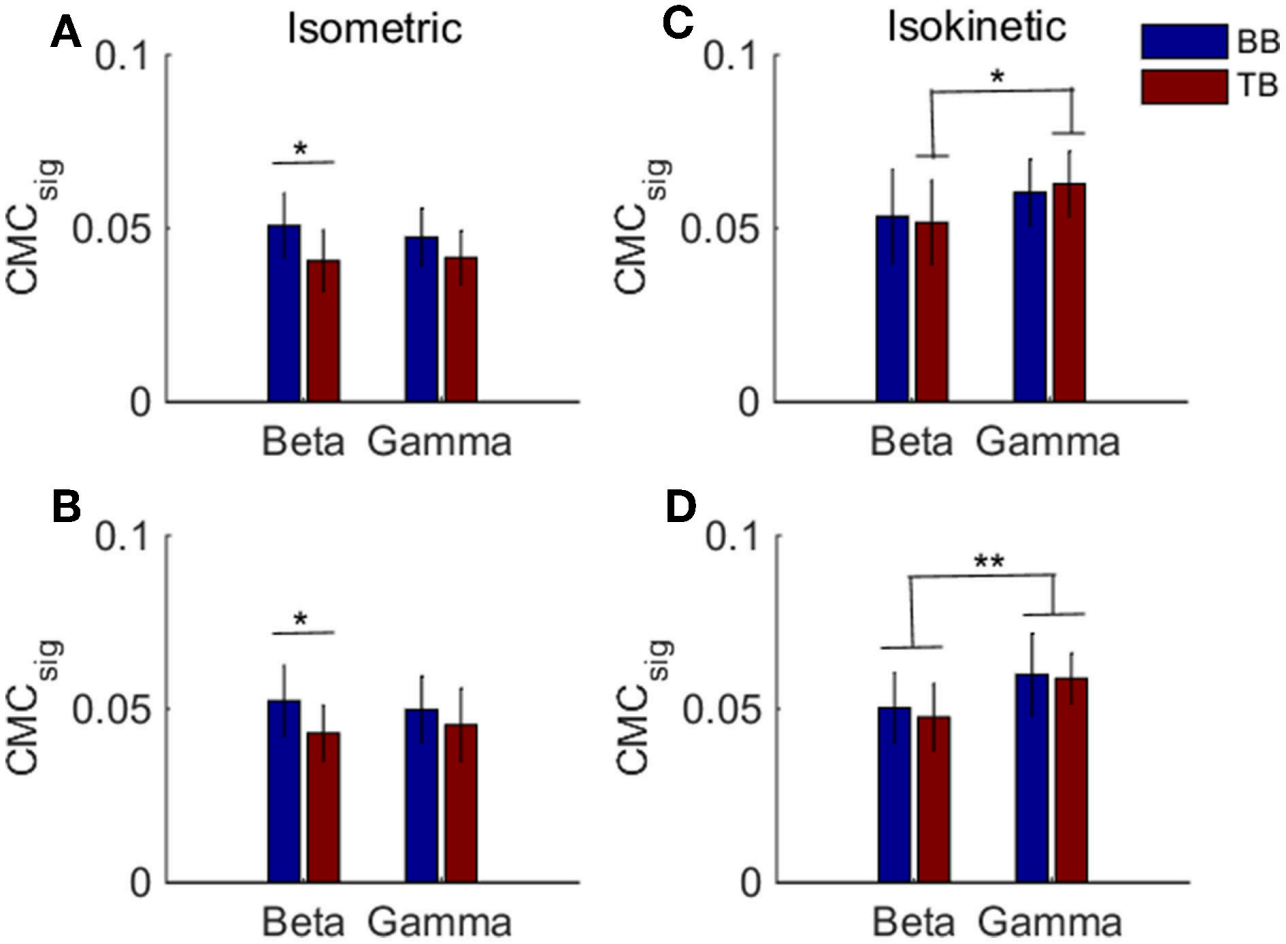
Grand averages of the *CMC*_*sig*_ values for BB and TB when muscles acted as agonist during the contraction stage of isometric exercise and isokinetic movement, in beta- and gamma-range, respectively. For isometric exercise, **(A,B)** show the *CMC*_*sig*_ values under C1 and C5 electrodes, respectively. For isokinetic movement, **(C,D)** show the *CMC*_*sig*_ values under C1 and C5 electrodes, respectively. We denoted the significance with the star mark. *p < 0.05 and **p < 0.01. Error bars show the standard error of the mean.

Previous results suggested that CMC acquired when muscles acted as agonist during contraction stage was more likely to reflect the corticospinal connection. To examine whether there was a significant difference in the BB-C1, TB-C1, BB-C5, and TB-C5 coherence during the muscles contraction stage of the tasks, we utilized a two-way ANOVA to assess the statistical significance in grand average of *CMC*_*sig*_ for independent variables frequency (beta or gamma) and muscles (BB or TB) in contraction stage of cyclically isokinetic movement and isometric exercise, respectively. For the *CMC*_*sig*_ values during isometric exercise, as shown in Figures 7A,B, a two-way ANOVA revealed that there were no significant interactions between the frequency bands and muscles under C1 [*F*_(1,36)_ = 0.559, *p* = 0.460] and C5 electrodes [*F*_(1,36)_ = 0.592, *p* = 0.447]. However, the *CMC*_*sig*_ value differed significantly between BB and TB muscles in beta-range under C1 [*F*_(1,36)_ = 6.372, *p* = 0.016] and C5 electrodes [*F*_(1,36)_ = 4.226, *p* = 0.047]. In beta-range, the *CMC*_*sig*_ value for BB muscle was obviously higher compared with TB muscle during isometric contraction stage. Similar results were observed for coherence in C1 and C5 channels. Additionally, for the *CMC*_*sig*_ values during isokinetic movement, as shown in Figures 7C,D, the effect of interaction between the frequency bands and muscles were also no significant under C1 [*F*_(1,36)_ = 0.313, *p* = 0.579] and C5 electrodes [*F*_(1,36)_ = 0.055, *p* = 0.815]. However, the *CMC*_*sig*_ value differed significantly between beta-range and gamma-range for TB muscle under C1 electrodes [*F*_(1,36)_ = 4.402, *p* = 0.043], and the main effect of frequency bands was significant for both muscles under C5 electrodes [*F*_(1,36)_ = 9.985, *p* = 0.003]. Compared to gamma-range, the *CMC*_*sig*_ values of the beta-range were lower in BB-C1 coherence. Similar results were shown in BB-C5 and TB-C5 coherence during isokinetic contraction stage. Unlike the isometric exercise, no significant difference in the *CMC*_*sig*_ value of muscles was observed in the isokinetic movement, either C1 electrode [*F*_(1,36)_ = 0.008, *p* = 0.930] or C5 electrode [*F*_(1,36)_ = 0.344, *p* = 0.561], which meant that the CMC of BB and TB muscles was approximately consistent when muscles acted as agonist during isokinetic movement.

## Discussion

In this study, we utilize the empirical wavelet coherence approach to calculate the significant coherence area in the time-frequency domain, and apply it in the tasks of isometric exercise and cyclically isokinetic movement. This study is the first to investigate the relationship between cortical-muscular functional connections in elbow flexion-extension stages during cyclically isokinetic movement and concludes the difference in the relationship between isometric exercise and isokinetic movement. The major findings are (i) the gamma-range coherence is significant higher during isokinetic movement compared with isometric exercise, (ii) significant CMC is maintained in the entire flexion-extension stage regardless the muscles contracting nature and prefers to contraction stage when muscles acted as agonist during isokinetic movement, and (iii) the CMC of extensor and flexor that act as agonist during contraction phase are explicitly consistent in cyclically isokinetic elbow movement.

### CMC During Isometric Exercise and Cyclically Isokinetic Movement

Through the comparison of *CMC*_*sig*_ value scalp-maps, we have found that significant coherence between upper arm muscles EMG signals and contralateral sensorimotor cortex EEG signals was observed in the beta- and gamma-range for both isometric and cyclically isokinetic movement. In accordance with the previous result (Gwin and Ferris, 2012a), beta-range coherence was significantly greater than gamma-range coherence for isometric exercises. We hypothesized that the significant coherence area of cyclically isokinetic movement in gamma-range would be higher than that of isometric exercise. The results of quantitative analysis of the *CMC*_*sig*_ values revealed the gamma-range coherence of isokinetic movement during the whole flexion and extension stages was greatly increased compared with that in isometric exercise. Additionally, the *CMC*_*sig*_ values were lower in beta-range compared with gamma-range for isokinetic condition, on the contrary, the *CMC*_*sig*_ values were significantly higher in beta-range than that in gamma-range during isometric exercise. Our research was consistent with the view of Omlor et al. (2007) that the most distinct coherence transferred to gamma-range under the condition of dynamic force. However, Omlor et al. (2007) only proved that coherence shifted to high frequency during dynamic force output in frequency-domain, ignoring the effect of time-domain. We validated the existence of coherence which transferred to higher frequencies by means of significant coherence areas proportion in time-frequency domain. Furthermore, our findings demonstrated that the motor cortical neuros were also sensitive within high force range, which was contrary to previous studies that lower-level forces were more sensitive to cortical neurons (Hepp-Reymond et al., 1989).

Marsden et al. (2000) proposed that when performing different tasks, coherence tended to shift to new frequencies, although the same muscles were involved. That means those neuronal groups contributed to a given action were characterized by their tendency to resonate at specific frequencies. Extending this view, Omlor et al. (2007) suggested the origin that the sensorimotor system would resonate at higher frequencies under dynamic force condition and oscillations of the higher frequency corticospinal network may promote rapid recalibration of the sensorimotor system required by dynamic force condition. However, joint movement had been neglected or absent in previous studies (Schoffelen et al., 2005; Omlor et al., 2007), and it could not be inferred to what extended the displacement change of limb caused the change of different frequencies in the results. In contrast, we clearly showed that CMC frequency shifted significantly to higher frequencies was related to joint displacement by continuous change of joint angle in isokinetic movement. It was not surprising that significant CMC was associated with beta-range in isometric exercise. Many studies had confirmed this result (Brown, 2000; Kristeva-Feige et al., 2002; Omlor et al., 2007), which was related to the inhibition of cortical activities during static force output.

### CMC for Flexors and Extensors During Flexion-Extension Stage

It has also been indicated that when performed isometric exercise and dynamic concentric plantar flexion with sufficient ankle angle and forces, the discharging rate of the motor unit was significantly higher during the dynamic plantar flexion, which shown that the nature of the contraction affected the recruitment of the motor unit (Kallio et al., 2013). Previous studies focused on sustained muscle contractions or single contraction form (Divekar and John, 2013; Lou et al., 2013; Hu et al., 2018), which neglected the participation of sensorimotor cortex in relaxed or low-contraction nature muscles. In our study, we considered the whole contraction and relaxation stages of flexors and extensors acting as agonist and antagonist in continuous flexion-extension stage. Isokinetic movement of the elbow joint was divided into cyclical movements and the complete cycle included contraction and relaxation state of muscles. Our findings showed significant coherence existed in the whole flexion-extension stage of muscles via the group average time-frequency maps of significant coherence. Additionally, compared to relaxation stage, the significant coherence was higher when muscles acted as agonist in contraction stage during cyclical isokinetic movement. This phenomenon was not observed in isometric exercise. These findings were supported by similar results related to dynamic increase in CMC during cyclical ankle flexion and extension movements (Yoshida et al., 2017). The authors of that study found that coherence increased cyclically between tibialis anterior and medial gastrocnemius muscles and midline primary motor cortex during the whole movement process through the simple bilateral exercise experiment of the feet.

Corticomuscluar coherence of the agonistic and antagonistic muscles was observed during sustained isometric elbow flexion (Bayram et al., 2015). Ushiyama et al. (2011) and Desmyttere et al. (2018) demonstrated that corticomuscular coherence was altered according to the contraction phases during isometric contractions. However, to our knowledge, no studies had explicitly quantified the relationship of coherence between flexors and extensors which acted as agonist during elbow isokinetic contraction stage. We hypothesized that CMC would be greater for flexor muscles than extensor muscles in contraction stage during the isokinetic cycle, since flexors had a stronger corticospinal connection. Contrary to the discovery of Bayram et al. (2015), we found that the CMC for extensor muscle was basically consistent with flexor muscle in contraction stage during cyclically isokinetic elbow movement, which proved that the result of the hypothesis was unsubstantiated. For the isometric contraction, the result of CMC was consistent with prior research that flexor muscles acted as agonist showed higher CMC value comparing with extensor muscles acted as agonist. Our finding suggested that extensors played an equally important role in contraction stage during isokinetic elbow movement, and differed from the effective of agonist in sustained isometric contraction.

## Conclusion

In this study, it is shown that significant coherence between EMG signals of flexors and extensors and contralateral sensorimotor cortex EEG signals was observed in the beta- and gamma-range for both isometric exercise and cyclically isokinetic movement. However, the gamma-range coherence for isokinetic movement was greatly increased compared with isometric exercise. On the other hand, significant CMC was maintained in the entire flexion-extension stage regardless the nature of muscles contraction, although dominant synchronization of cortical oscillation and muscular activity principally resonated in contraction stage. Furthermore, the CMC for upper arm extensor muscle was explicitly consistent with that for flexor muscle in contraction stage when muscles acted as agonist during cyclically isokinetic elbow movement. These results suggest that corticomuscular coherence could be dynamically modulated as well as selective by cognitive demands of the body, and reveal the time-varying mechanism of the synchronous motor oscillation during dynamic movement.

## Ethics Statement

This study was carried out in accordance with the recommendations of Ethical Committee of Shanghai Jiao Tong University with written informed consent from all subjects. All subjects gave written informed consent in accordance with the Declaration of Helsinki. The protocol was approved by the Ethical Committee of Shanghai Jiao Tong University.

## Author Contributions

HL, JL, YS, and JZ contributed conception and design of the study. JL organized the database and performed the statistical analysis, wrote the first draft of the manuscript. YS and JZ wrote sections of the manuscript. All authors contributed to manuscript revision, read and approved the submitted version.

### Conflict of Interest Statement

The authors declare that the research was conducted in the absence of any commercial or financial relationships that could be construed as a potential conflict of interest.
